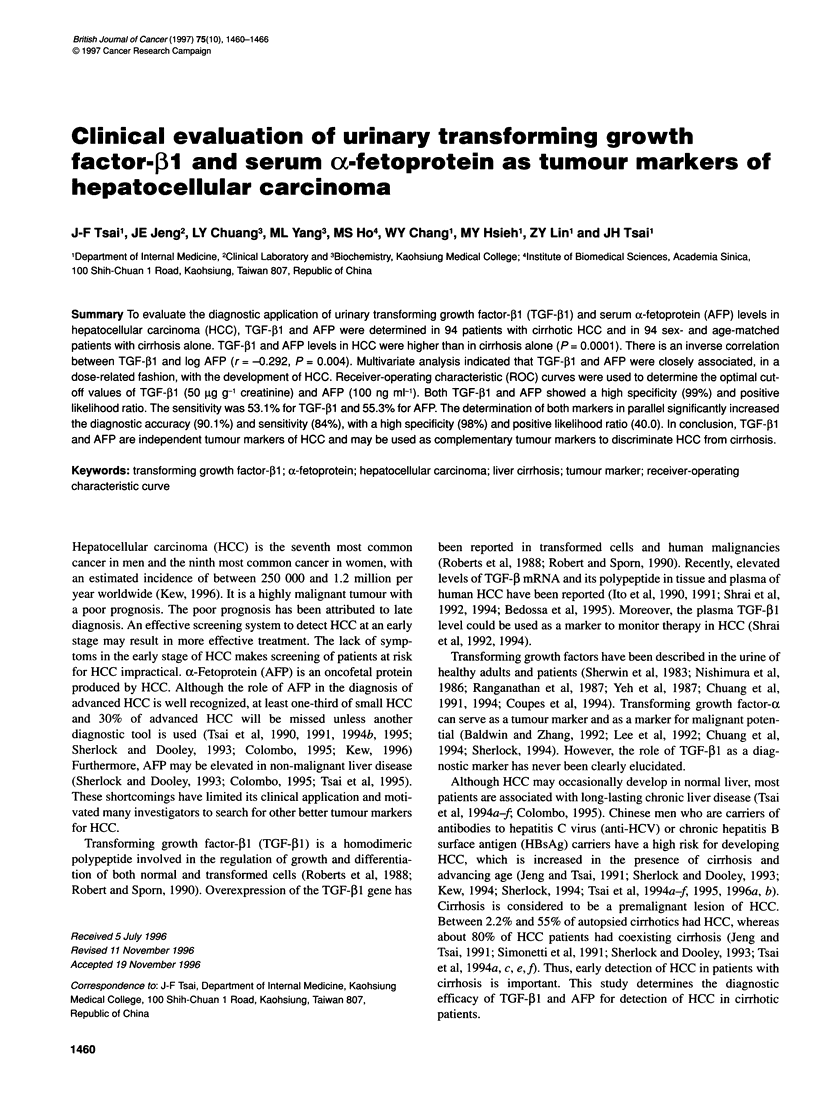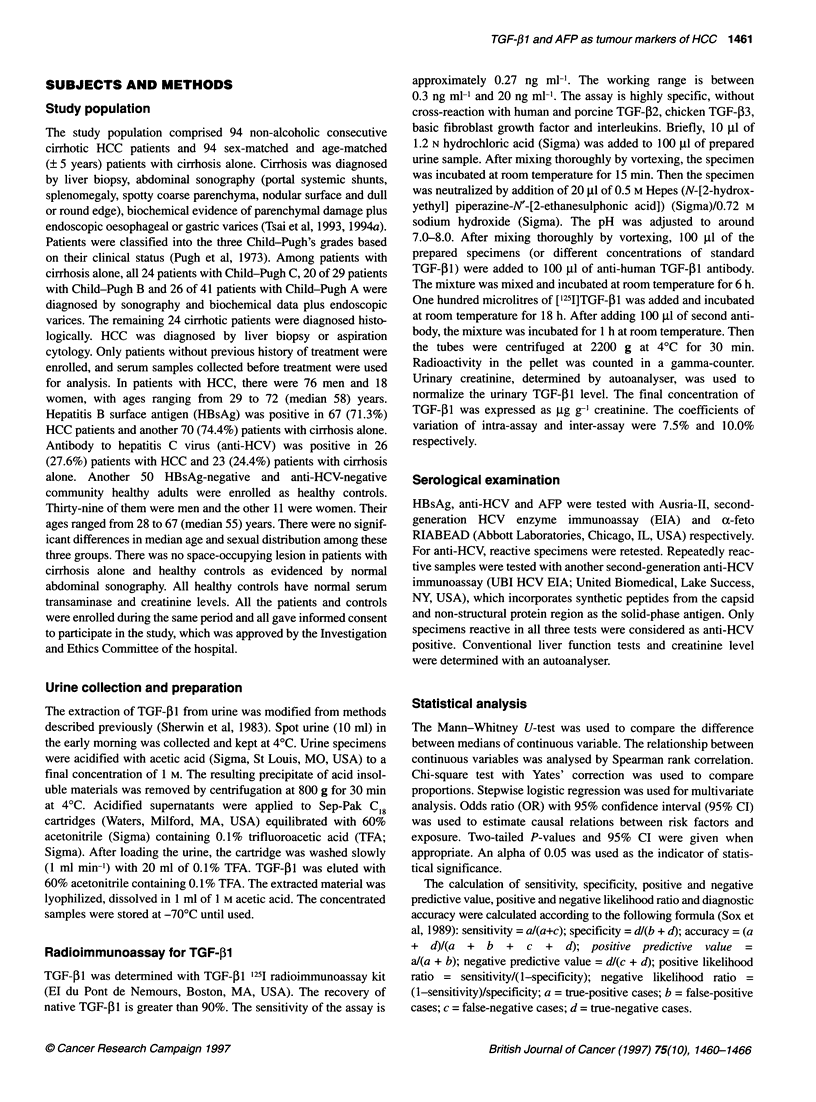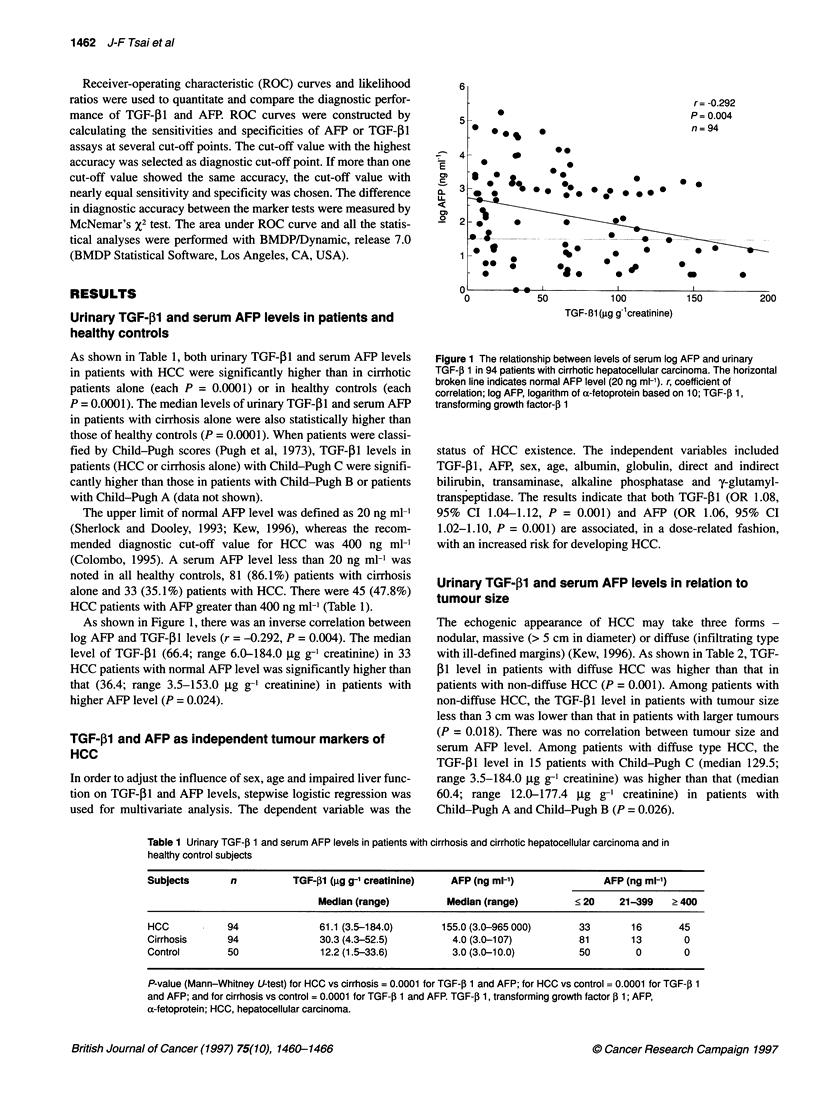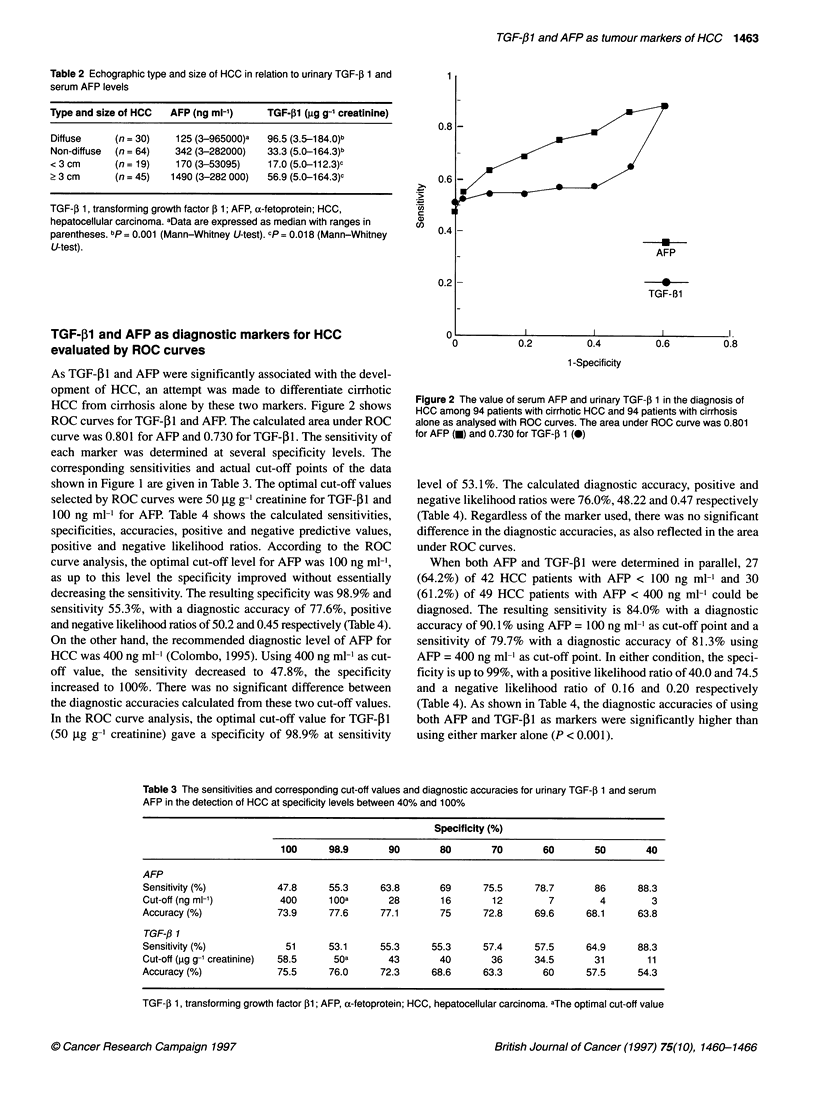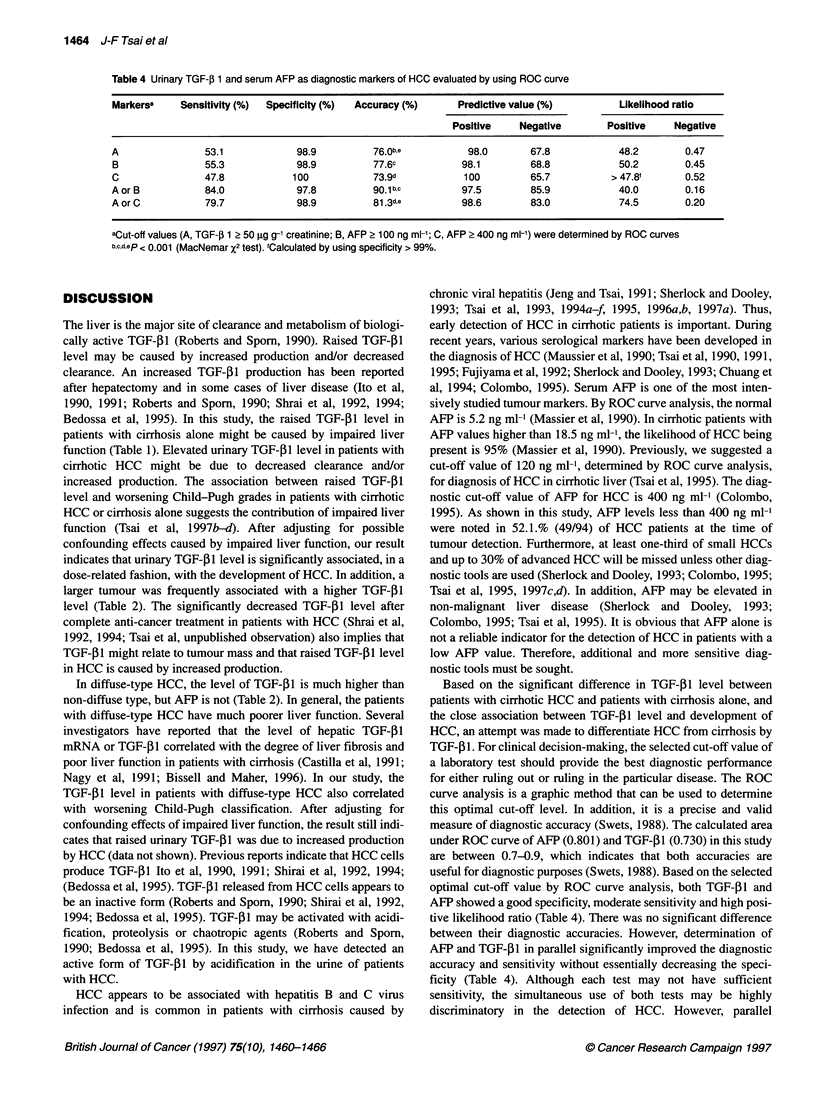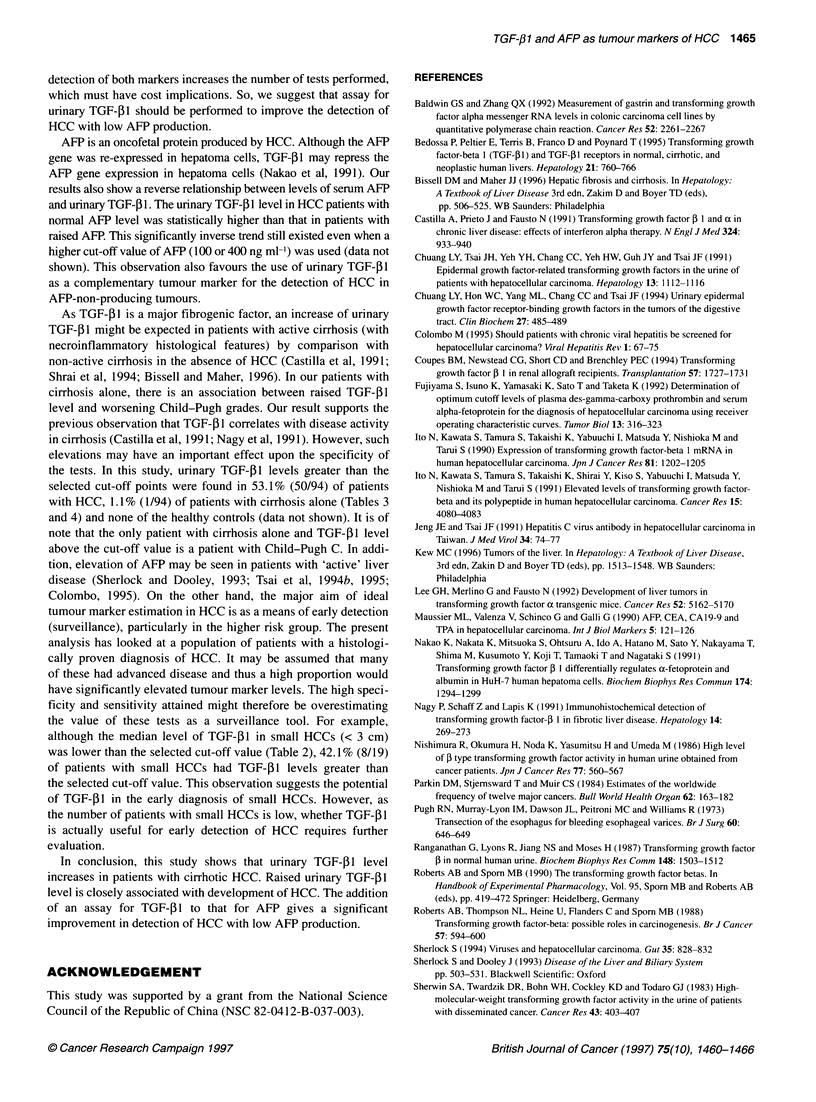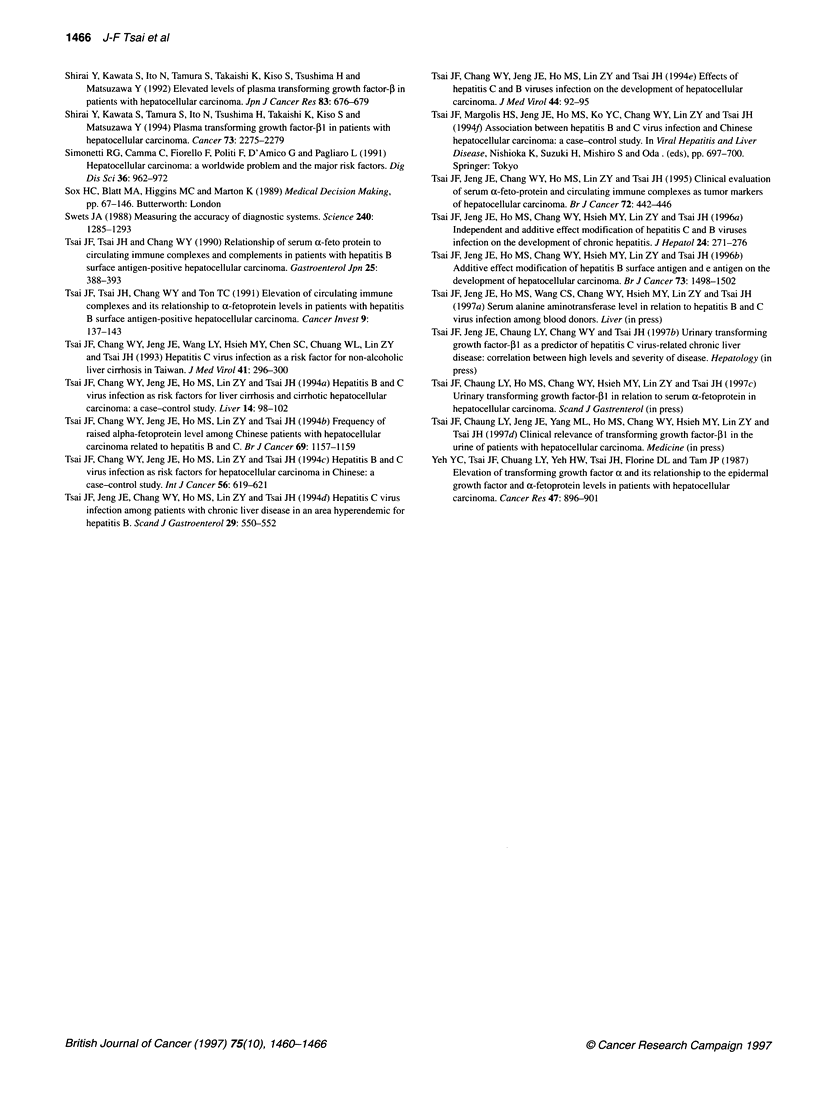# Clinical evaluation of urinary transforming growth factor-beta1 and serum alpha-fetoprotein as tumour markers of hepatocellular carcinoma.

**DOI:** 10.1038/bjc.1997.250

**Published:** 1997

**Authors:** J. F. Tsai, J. E. Jeng, L. Y. Chuang, M. L. Yang, M. S. Ho, W. Y. Chang, M. Y. Hsieh, Z. Y. Lin, J. H. Tsai

**Affiliations:** Department of Internal Medicine, Kaohsiung Medical College, Taiwan, Republic of China.

## Abstract

To evaluate the diagnostic application of urinary transforming growth factor-beta1 (TGF-beta1) and serum alpha-fetoprotein (AFP) levels in hepatocellular carcinoma (HCC), TGF-beta1 and AFP were determined in 94 patients with cirrhotic HCC and in 94 sex- and age-matched patients with cirrhosis alone. TGF-beta1 and AFP levels in HCC were higher than in cirrhosis alone (P = 0.0001). There is an inverse correlation between TGF-beta1 and log AFP (r = -0.292, P = 0.004). Multivariate analysis indicated that TGF-beta1 and AFP were closely associated, in a dose-related fashion, with the development of HCC. Receiver-operating characteristic (ROC) curves were used to determine the optimal cut-off values of TGF-beta1 (50 microg g(-1) creatinine) and AFP (100 ng ml(-1)). Both TGF-beta1 and AFP showed a high specificity (99%) and positive likelihood ratio. The sensitivity was 53.1% for TGF-beta1 and 55.3% for AFP. The determination of both markers in parallel significantly increased the diagnostic accuracy (90.1%) and sensitivity (84%), with a high specificity (98%) and positive likelihood ratio (40.0). In conclusion, TGF-beta1 and AFP are independent tumour markers of HCC and may be used as complementary tumour markers to discriminate HCC from cirrhosis.


*British Journal of Cancer* (1997) **75**(10), 1460–1466
© 1997 Cancer Research Campaign

### Clinical evaluation of urinary transforming growth factor-β1 and serum α-fetoprotein as tumour markers of hepatocellular carcinoma

**J-F Tsai[1], JE Jeng[2], LY Chuang[3], ML Yang[3], MS Ho[4], WY Chang[1], MY Hsieh[1], ZY Lin[1] and JH Tsai[1]**

[1]Department of Internal Medicine, [2]Clinical Laboratory and [3]Biochemistry, Kaohsiung Medical College; [4]Institute of Biomedical Sciences, Academia Sinica, 100 Shih-Chuan 1 Road, Kaohsiung, Taiwan 807, Republic of China

**Summary** To evaluate the diagnostic application of urinary transforming growth factor-β1 (TGF-β1) and serum α-fetoprotein (AFP) levels in hepatocellular carcinoma (HCC), TGF-β1 and AFP were determined in 94 patients with cirrhotic HCC and in 94 sex- and age-matched patients with cirrhosis alone. TGF-β1 and AFP levels in HCC were higher than in cirrhosis alone ($P = 0.0001$). There is an inverse correlation between TGF-β1 and log AFP ($r = -0.292$, $P = 0.004$). Multivariate analysis indicated that TGF-β1 and AFP were closely associated, in a dose-related fashion, with the development of HCC. Receiver-operating characteristic (ROC) curves were used to determine the optimal cut-off values of TGF-β1 (50 μg $g^{-1}$ creatinine) and AFP (100 ng $ml^{-1}$). Both TGF-β1 and AFP showed a high specificity (99%) and positive likelihood ratio. The sensitivity was 53.1% for TGF-β1 and 55.3% for AFP. The determination of both markers in parallel significantly increased the diagnostic accuracy (90.1%) and sensitivity (84%), with a high specificity (98%) and positive likelihood ratio (40.0). In conclusion, TGF-β1 and AFP are independent tumour markers of HCC and may be used as complementary tumour markers to discriminate HCC from cirrhosis.

**Keywords:** transforming growth factor-β1; α-fetoprotein; hepatocellular carcinoma; liver cirrhosis; tumour marker; receiver-operating characteristic curve

Hepatocellular carcinoma (HCC) is the seventh most common cancer in men and the ninth most common cancer in women, with an estimated incidence of between 250 000 and 1.2 million per year worldwide (Kew, 1996). It is a highly malignant tumour with a poor prognosis. The poor prognosis has been attributed to late diagnosis. An effective screening system to detect HCC at an early stage may result in more effective treatment. The lack of symptoms in the early stage of HCC makes screening of patients at risk for HCC impractical. α-Fetoprotein (AFP) is an oncofetal protein produced by HCC. Although the role of AFP in the diagnosis of advanced HCC is well recognized, at least one-third of small HCC and 30% of advanced HCC will be missed unless another diagnostic tool is used (Tsai et al, 1990, 1991, 1994*b*, 1995; Sherlock and Dooley, 1993; Colombo, 1995; Kew, 1996) Furthermore, AFP may be elevated in non-malignant liver disease (Sherlock and Dooley, 1993; Colombo, 1995; Tsai et al, 1995). These shortcomings have limited its clinical application and motivated many investigators to search for other better tumour markers for HCC.

Transforming growth factor-β1 (TGF-β1) is a homodimeric polypeptide involved in the regulation of growth and differentiation of both normal and transformed cells (Roberts et al, 1988; Robert and Sporn, 1990). Overexpression of the TGF-β1 gene has been reported in transformed cells and human malignancies (Roberts et al, 1988; Robert and Sporn, 1990). Recently, elevated levels of TGF-β mRNA and its polypeptide in tissue and plasma of human HCC have been reported (Ito et al, 1990, 1991; Shrai et al, 1992, 1994; Bedossa et al, 1995). Moreover, the plasma TGF-β1 level could be used as a marker to monitor therapy in HCC (Shrai et al, 1992, 1994).

Transforming growth factors have been described in the urine of healthy adults and patients (Sherwin et al, 1983; Nishimura et al, 1986; Ranganathan et al, 1987; Yeh et al, 1987; Chuang et al, 1991, 1994; Coupes et al, 1994). Transforming growth factor-α can serve as a tumour marker and as a marker for malignant potential (Baldwin and Zhang, 1992; Lee et al, 1992; Chuang et al, 1994; Sherlock, 1994). However, the role of TGF-β1 as a diagnostic marker has never been clearly elucidated.

Although HCC may occasionally develop in normal liver, most patients are associated with long-lasting chronic liver disease (Tsai et al, 1994*a–f*; Colombo, 1995). Chinese men who are carriers of antibodies to hepatitis C virus (anti-HCV) or chronic hepatitis B surface antigen (HBsAg) carriers have a high risk for developing HCC, which is increased in the presence of cirrhosis and advancing age (Jeng and Tsai, 1991; Sherlock and Dooley, 1993; Kew, 1994; Sherlock, 1994; Tsai et al, 1994*a–f*, 1995, 1996*a*, *b*). Cirrhosis is considered to be a premalignant lesion of HCC. Between 2.2% and 55% of autopsied cirrhotics had HCC, whereas about 80% of HCC patients had coexisting cirrhosis (Jeng and Tsai, 1991; Simonetti et al, 1991; Sherlock and Dooley, 1993; Tsai et al, 1994*a*, *c*, *e*, *f*). Thus, early detection of HCC in patients with cirrhosis is important. This study determines the diagnostic efficacy of TGF-β1 and AFP for detection of HCC in cirrhotic patients.

*Received 5 July 1996*
*Revised 11 November 1996*
*Accepted 19 November 1996*

*Correspondence to*: J-F Tsai, Department of Internal Medicine, Kaohsiung Medical College, 100 Shih-Chuan 1 Road, Kaohsiung, Taiwan 807, Republic of China

### SUBJECTS AND METHODS

### Study population

The study population comprised 94 non-alcoholic consecutive cirrhotic HCC patients and 94 sex-matched and age-matched (± 5 years) patients with cirrhosis alone. Cirrhosis was diagnosed by liver biopsy, abdominal sonography (portal systemic shunts, splenomegaly, spotty coarse parenchyma, nodular surface and dull or round edge), biochemical evidence of parenchymal damage plus endoscopic oesophageal or gastric varices (Tsai et al, 1993, 1994*a*). Patients were classified into the three Child–Pugh's grades based on their clinical status (Pugh et al, 1973). Among patients with cirrhosis alone, all 24 patients with Child–Pugh C, 20 of 29 patients with Child–Pugh B and 26 of 41 patients with Child–Pugh A were diagnosed by sonography and biochemical data plus endoscopic varices. The remaining 24 cirrhotic patients were diagnosed histologically. HCC was diagnosed by liver biopsy or aspiration cytology. Only patients without previous history of treatment were enrolled, and serum samples collected before treatment were used for analysis. In patients with HCC, there were 76 men and 18 women, with ages ranging from 29 to 72 (median 58) years. Hepatitis B surface antigen (HBsAg) was positive in 67 (71.3%) HCC patients and another 70 (74.4%) patients with cirrhosis alone. Antibody to hepatitis C virus (anti-HCV) was positive in 26 (27.6%) patients with HCC and 23 (24.4%) patients with cirrhosis alone. Another 50 HBsAg-negative and anti-HCV-negative community healthy adults were enrolled as healthy controls. Thirty-nine of them were men and the other 11 were women. Their ages ranged from 28 to 67 (median 55) years. There were no significant differences in median age and sexual distribution among these three groups. There was no space-occupying lesion in patients with cirrhosis alone and healthy controls as evidenced by normal abdominal sonography. All healthy controls have normal serum transaminase and creatinine levels. All the patients and controls were enrolled during the same period and all gave informed consent to participate in the study, which was approved by the Investigation and Ethics Committee of the hospital.

### Urine collection and preparation

The extraction of TGF-β1 from urine was modified from methods described previously (Sherwin et al, 1983). Spot urine (10 ml) in the early morning was collected and kept at 4°C. Urine specimens were acidified with acetic acid (Sigma, St Louis, MO, USA) to a final concentration of 1 M. The resulting precipitate of acid insoluble materials was removed by centrifugation at 800 **g** for 30 min at 4°C. Acidified supernatants were applied to Sep-Pak $C_{18}$ cartridges (Waters, Milford, MA, USA) equilibrated with 60% acetonitrile (Sigma) containing 0.1% trifluoroacetic acid (TFA; Sigma). After loading the urine, the cartridge was washed slowly (1 ml $min^{-1}$) with 20 ml of 0.1% TFA. TGF-β1 was eluted with 60% acetonitrile containing 0.1% TFA. The extracted material was lyophilized, dissolved in 1 ml of 1 M acetic acid. The concentrated samples were stored at –70°C until used.

### Radioimmunoassay for TGF-β1

TGF-β1 was determined with TGF-β1 $^{125}I$ radioimmunoassay kit (EI du Pont de Nemours, Boston, MA, USA). The recovery of native TGF-β1 is greater than 90%. The sensitivity of the assay is approximately 0.27 ng $ml^{-1}$. The working range is between 0.3 ng $ml^{-1}$ and 20 ng $ml^{-1}$. The assay is highly specific, without cross-reaction with human and porcine TGF-β2, chicken TGF-β3, basic fibroblast growth factor and interleukins. Briefly, 10 μl of 1.2 N hydrochloric acid (Sigma) was added to 100 μl of prepared urine sample. After mixing thoroughly by vortexing, the specimen was incubated at room temperature for 15 min. Then the specimen was neutralized by addition of 20 μl of 0.5 M Hepes (*N*-[2-hydroxyethyl] piperazine-*N'*-[2-ethanesulphonic acid]) (Sigma)/0.72 M sodium hydroxide (Sigma). The pH was adjusted to around 7.0–8.0. After mixing thoroughly by vortexing, 100 μl of the prepared specimens (or different concentrations of standard TGF-β1) were added to 100 μl of anti-human TGF-β1 antibody. The mixture was mixed and incubated at room temperature for 6 h. One hundred microlitres of [$^{125}I$]TGF-β1 was added and incubated at room temperature for 18 h. After adding 100 μl of second antibody, the mixture was incubated for 1 h at room temperature. Then the tubes were centrifuged at 2200 **g** at 4°C for 30 min. Radioactivity in the pellet was counted in a gamma-counter. Urinary creatinine, determined by autoanalyser, was used to normalize the urinary TGF-β1 level. The final concentration of TGF-β1 was expressed as μg $g^{-1}$ creatinine. The coefficients of variation of intra-assay and inter-assay were 7.5% and 10.0% respectively.

### Serological examination

HBsAg, anti-HCV and AFP were tested with Ausria-II, second-generation HCV enzyme immunoassay (EIA) and α-feto RIABEAD (Abbott Laboratories, Chicago, IL, USA) respectively. For anti-HCV, reactive specimens were retested. Repeatedly reactive samples were tested with another second-generation anti-HCV immunoassay (UBI HCV EIA; United Biomedical, Lake Success, NY, USA), which incorporates synthetic peptides from the capsid and non-structural protein region as the solid-phase antigen. Only specimens reactive in all three tests were considered as anti-HCV positive. Conventional liver function tests and creatinine level were determined with an autoanalyser.

### Statistical analysis

The Mann–Whitney *U*-test was used to compare the difference between medians of continuous variable. The relationship between continuous variables was analysed by Spearman rank correlation. Chi-square test with Yates' correction was used to compare proportions. Stepwise logistic regression was used for multivariate analysis. Odds ratio (OR) with 95% confidence interval (95% CI) was used to estimate causal relations between risk factors and exposure. Two-tailed *P*-values and 95% CI were given when appropriate. An alpha of 0.05 was used as the indicator of statistical significance.

The calculation of sensitivity, specificity, positive and negative predictive value, positive and negative likelihood ratio and diagnostic accuracy were calculated according to the following formula (Sox et al, 1989): sensitivity = $a/(a+c)$; specificity = $d/(b + d)$; accuracy = $(a + d)/(a + b + c + d)$; *positive predictive value* = $a/(a + b)$; negative predictive value = $d/(c + d)$; positive likelihood ratio = sensitivity/(1–specificity); negative likelihood ratio = (1–sensitivity)/specificity; $a$ = true-positive cases; $b$ = false-positive cases; $c$ = false-negative cases; $d$ = true-negative cases.

© Cancer Research Campaign 1997

Receiver-operating characteristic (ROC) curves and likelihood ratios were used to quantitate and compare the diagnostic performance of TGF-β1 and AFP. ROC curves were constructed by calculating the sensitivities and specificities of AFP or TGF-β1 assays at several cut-off points. The cut-off value with the highest accuracy was selected as diagnostic cut-off point. If more than one cut-off value showed the same accuracy, the cut-off value with nearly equal sensitivity and specificity was chosen. The difference in diagnostic accuracy between the marker tests were measured by McNemar's $\chi^2$ test. The area under ROC curve and all the statistical analyses were performed with BMDP/Dynamic, release 7.0 (BMDP Statistical Software, Los Angeles, CA, USA).

### RESULTS

### Urinary TGF-β1 and serum AFP levels in patients and healthy controls

As shown in Table 1, both urinary TGF-β1 and serum AFP levels in patients with HCC were significantly higher than in cirrhotic patients alone (each $P$ = 0.0001) or in healthy controls (each $P$ = 0.0001). The median levels of urinary TGF-β1 and serum AFP in patients with cirrhosis alone were also statistically higher than those of healthy controls ($P$ = 0.0001). When patients were classified by Child–Pugh scores (Pugh et al, 1973), TGF-β1 levels in patients (HCC or cirrhosis alone) with Child–Pugh C were significantly higher than those in patients with Child–Pugh B or patients with Child–Pugh A (data not shown).

The upper limit of normal AFP level was defined as 20 ng ml$^{-1}$ (Sherlock and Dooley, 1993; Kew, 1996), whereas the recommended diagnostic cut-off value for HCC was 400 ng ml$^{-1}$ (Colombo, 1995). A serum AFP level less than 20 ng ml$^{-1}$ was noted in all healthy controls, 81 (86.1%) patients with cirrhosis alone and 33 (35.1%) patients with HCC. There were 45 (47.8%) HCC patients with AFP greater than 400 ng ml$^{-1}$ (Table 1).

As shown in Figure 1, there was an inverse correlation between log AFP and TGF-β1 levels ($r$ = –0.292, $P$ = 0.004). The median level of TGF-β1 (66.4; range 6.0–184.0 μg g$^{-1}$ creatinine) in 33 HCC patients with normal AFP level was significantly higher than that (36.4; range 3.5–153.0 μg g$^{-1}$ creatinine) in patients with higher AFP level ($P$ = 0.024).

### TGF-β1 and AFP as independent tumour markers of HCC

In order to adjust the influence of sex, age and impaired liver function on TGF-β1 and AFP levels, stepwise logistic regression was used for multivariate analysis. The dependent variable was the status of HCC existence. The independent variables included TGF-β1, AFP, sex, age, albumin, globulin, direct and indirect bilirubin, transaminase, alkaline phosphatase and γ-glutamyl-transpeptidase. The results indicate that both TGF-β1 (OR 1.08, 95% CI 1.04–1.12, $P$ = 0.001) and AFP (OR 1.06, 95% CI 1.02–1.10, $P$ = 0.001) are associated, in a dose-related fashion, with an increased risk for developing HCC.

**Figure 1** The relationship between levels of serum log AFP and urinary TGF-β 1 in 94 patients with cirrhotic hepatocellular carcinoma. The horizontal broken line indicates normal AFP level (20 ng ml$^{-1}$). $r$, coefficient of correlation; log AFP, logarithm of α-fetoprotein based on 10; TGF-β 1, transforming growth factor-β 1

### Urinary TGF-β1 and serum AFP levels in relation to tumour size

The echogenic appearance of HCC may take three forms – nodular, massive (> 5 cm in diameter) or diffuse (infiltrating type with ill-defined margins) (Kew, 1996). As shown in Table 2, TGF-β1 level in patients with diffuse HCC was higher than that in patients with non-diffuse HCC ($P$ = 0.001). Among patients with non-diffuse HCC, the TGF-β1 level in patients with tumour size less than 3 cm was lower than that in patients with larger tumours ($P$ = 0.018). There was no correlation between tumour size and serum AFP level. Among patients with diffuse type HCC, the TGF-β1 level in 15 patients with Child–Pugh C (median 129.5; range 3.5–184.0 μg g$^{-1}$ creatinine) was higher than that (median 60.4; range 12.0–177.4 μg g$^{-1}$ creatinine) in patients with Child–Pugh A and Child–Pugh B ($P$ = 0.026).

**Table 1** Urinary TGF-β 1 and serum AFP levels in patients with cirrhosis and cirrhotic hepatocellular carcinoma and in healthy control subjects

| Subjects | n | TGF-β1 (μg g$^{-1}$ creatinine) | AFP (ng ml$^{-1}$) | AFP (ng ml$^{-1}$) | | |
|---|---|---|---|---|---|---|
| | | Median (range) | Median (range) | ≤ 20 | 21–399 | ≥ 400 |
| HCC | 94 | 61.1 (3.5–184.0) | 155.0 (3.0–965 000) | 33 | 16 | 45 |
| Cirrhosis | 94 | 30.3 (4.3–52.5) | 4.0 (3.0–107) | 81 | 13 | 0 |
| Control | 50 | 12.2 (1.5–33.6) | 3.0 (3.0–10.0) | 50 | 0 | 0 |

$P$-value (Mann–Whitney $U$-test) for HCC vs cirrhosis = 0.0001 for TGF-β 1 and AFP; for HCC vs control = 0.0001 for TGF-β 1 and AFP; and for cirrhosis vs control = 0.0001 for TGF-β 1 and AFP. TGF-β 1, transforming growth factor β 1; AFP, α-fetoprotein; HCC, hepatocellular carcinoma.

© Cancer Research Campaign 1997

**Table 2** Echographic type and size of HCC in relation to urinary TGF-β 1 and serum AFP levels

| Type and size of HCC | | AFP (ng ml$^{-1}$) | TGF-β1 (μg g$^{-1}$ creatinine) |
|---|---|---|---|
| Diffuse | (*n* = 30) | 125 (3–965000)[a] | 96.5 (3.5–184.0)[b] |
| Non-diffuse | (*n* = 64) | 342 (3–282000) | 33.3 (5.0–164.3)[b] |
| < 3 cm | (*n* = 19) | 170 (3–53095) | 17.0 (5.0–112.3)[c] |
| ≥ 3 cm | (*n* = 45) | 1490 (3–282 000) | 56.9 (5.0–164.3)[c] |

TGF-β 1, transforming growth factor β 1; AFP, α-fetoprotein; HCC, hepatocellular carcinoma. [a]Data are expressed as median with ranges in parentheses. [b]$P$ = 0.001 (Mann–Whitney *U*-test). [c]$P$ = 0.018 (Mann–Whitney *U*-test).

### TGF-β1 and AFP as diagnostic markers for HCC evaluated by ROC curves

As TGF-β1 and AFP were significantly associated with the development of HCC, an attempt was made to differentiate cirrhotic HCC from cirrhosis alone by these two markers. Figure 2 shows ROC curves for TGF-β1 and AFP. The calculated area under ROC curve was 0.801 for AFP and 0.730 for TGF-β1. The sensitivity of each marker was determined at several specificity levels. The corresponding sensitivities and actual cut-off points of the data shown in Figure 1 are given in Table 3. The optimal cut-off values selected by ROC curves were 50 μg g$^{-1}$ creatinine for TGF-β1 and 100 ng ml$^{-1}$ for AFP. Table 4 shows the calculated sensitivities, specificities, accuracies, positive and negative predictive values, positive and negative likelihood ratios. According to the ROC curve analysis, the optimal cut-off level for AFP was 100 ng ml$^{-1}$, as up to this level the specificity improved without essentially decreasing the sensitivity. The resulting specificity was 98.9% and sensitivity 55.3%, with a diagnostic accuracy of 77.6%, positive and negative likelihood ratios of 50.2 and 0.45 respectively (Table 4). On the other hand, the recommended diagnostic level of AFP for HCC was 400 ng ml$^{-1}$ (Colombo, 1995). Using 400 ng ml$^{-1}$ as cut-off value, the sensitivity decreased to 47.8%, the specificity increased to 100%. There was no significant difference between the diagnostic accuracies calculated from these two cut-off values. In the ROC curve analysis, the optimal cut-off value for TGF-β1 (50 μg g$^{-1}$ creatinine) gave a specificity of 98.9% at sensitivity

**Figure 2** The value of serum AFP and urinary TGF-β 1 in the diagnosis of HCC among 94 patients with cirrhotic HCC and 94 patients with cirrhosis alone as analysed with ROC curves. The area under ROC curve was 0.801 for AFP (■) and 0.730 for TGF-β 1 (●)

level of 53.1%. The calculated diagnostic accuracy, positive and negative likelihood ratios were 76.0%, 48.22 and 0.47 respectively (Table 4). Regardless of the marker used, there was no significant difference in the diagnostic accuracies, as also reflected in the area under ROC curves.

When both AFP and TGF-β1 were determined in parallel, 27 (64.2%) of 42 HCC patients with AFP < 100 ng ml$^{-1}$ and 30 (61.2%) of 49 HCC patients with AFP < 400 ng ml$^{-1}$ could be diagnosed. The resulting sensitivity is 84.0% with a diagnostic accuracy of 90.1% using AFP = 100 ng ml$^{-1}$ as cut-off point and a sensitivity of 79.7% with a diagnostic accuracy of 81.3% using AFP = 400 ng ml$^{-1}$ as cut-off point. In either condition, the specificity is up to 99%, with a positive likelihood ratio of 40.0 and 74.5 and a negative likelihood ratio of 0.16 and 0.20 respectively (Table 4). As shown in Table 4, the diagnostic accuracies of using both AFP and TGF-β1 as markers were significantly higher than using either marker alone ($P$ < 0.001).

**Table 3** The sensitivities and corresponding cut-off values and diagnostic accuracies for urinary TGF-β 1 and serum AFP in the detection of HCC at specificity levels between 40% and 100%

| | Specificity (%) | | | | | | | |
|---|---|---|---|---|---|---|---|---|
| | 100 | 98.9 | 90 | 80 | 70 | 60 | 50 | 40 |
| *AFP* | | | | | | | | |
| Sensitivity (%) | 47.8 | 55.3 | 63.8 | 69 | 75.5 | 78.7 | 86 | 88.3 |
| Cut-off (ng ml$^{-1}$) | 400 | 100[a] | 28 | 16 | 12 | 7 | 4 | 3 |
| Accuracy (%) | 73.9 | 77.6 | 77.1 | 75 | 72.8 | 69.6 | 68.1 | 63.8 |
| *TGF-β 1* | | | | | | | | |
| Sensitivity (%) | 51 | 53.1 | 55.3 | 55.3 | 57.4 | 57.5 | 64.9 | 88.3 |
| Cut-off (μg g$^{-1}$ creatinine) | 58.5 | 50[a] | 43 | 40 | 36 | 34.5 | 31 | 11 |
| Accuracy (%) | 75.5 | 76.0 | 72.3 | 68.6 | 63.3 | 60 | 57.5 | 54.3 |

TGF-β 1, transforming growth factor β1; AFP, α-fetoprotein; HCC, hepatocellular carcinoma. [a]The optimal cut-off value

**Table 4** Urinary TGF-β 1 and serum AFP as diagnostic markers of HCC evaluated by using ROC curve

| Markers[a] | Sensitivity (%) | Specificity (%) | Accuracy (%) | Predictive value (%) | | Likelihood ratio | |
|---|---|---|---|---|---|---|---|
| | | | | Positive | Negative | Positive | Negative |
| A | 53.1 | 98.9 | 76.0[b,e] | 98.0 | 67.8 | 48.2 | 0.47 |
| B | 55.3 | 98.9 | 77.6[c] | 98.1 | 68.8 | 50.2 | 0.45 |
| C | 47.8 | 100 | 73.9[d] | 100 | 65.7 | > 47.8[f] | 0.52 |
| A or B | 84.0 | 97.8 | 90.1[b,c] | 97.5 | 85.9 | 40.0 | 0.16 |
| A or C | 79.7 | 98.9 | 81.3[d,e] | 98.6 | 83.0 | 74.5 | 0.20 |

[a]Cut-off values (A, TGF-β 1 ≥ 50 μg $g^{-1}$ creatinine; B, AFP ≥ 100 ng $ml^{-1}$; C, AFP ≥ 400 ng $ml^{-1}$) were determined by ROC curves [b,c,d,e]$P < 0.001$ (MacNemar $\chi^2$ test). [f]Calculated by using specificity > 99%.

### DISCUSSION

The liver is the major site of clearance and metabolism of biologically active TGF-β1 (Roberts and Sporn, 1990). Raised TGF-β1 level may be caused by increased production and/or decreased clearance. An increased TGF-β1 production has been reported after hepatectomy and in some cases of liver disease (Ito et al, 1990, 1991; Roberts and Sporn, 1990; Shrai et al, 1992, 1994; Bedossa et al, 1995). In this study, the raised TGF-β1 level in patients with cirrhosis alone might be caused by impaired liver function (Table 1). Elevated urinary TGF-β1 level in patients with cirrhotic HCC might be due to decreased clearance and/or increased production. The association between raised TGF-β1 level and worsening Child–Pugh grades in patients with cirrhotic HCC or cirrhosis alone suggests the contribution of impaired liver function (Tsai et al, 1997*b–d*). After adjusting for possible confounding effects caused by impaired liver function, our result indicates that urinary TGF-β1 level is significantly associated, in a dose-related fashion, with the development of HCC. In addition, a larger tumour was frequently associated with a higher TGF-β1 level (Table 2). The significantly decreased TGF-β1 level after complete anti-cancer treatment in patients with HCC (Shrai et al, 1992, 1994; Tsai et al, unpublished observation) also implies that TGF-β1 might relate to tumour mass and that raised TGF-β1 level in HCC is caused by increased production.

In diffuse-type HCC, the level of TGF-β1 is much higher than non-diffuse type, but AFP is not (Table 2). In general, the patients with diffuse-type HCC have much poorer liver function. Several investigators have reported that the level of hepatic TGF-β1 mRNA or TGF-β1 correlated with the degree of liver fibrosis and poor liver function in patients with cirrhosis (Castilla et al, 1991; Nagy et al, 1991; Bissell and Maher, 1996). In our study, the TGF-β1 level in patients with diffuse-type HCC also correlated with worsening Child-Pugh classification. After adjusting for confounding effects of impaired liver function, the result still indicates that raised urinary TGF-β1 was due to increased production by HCC (data not shown). Previous reports indicate that HCC cells produce TGF-β1 Ito et al, 1990, 1991; Shirai et al, 1992, 1994; (Bedossa et al, 1995). TGF-β1 released from HCC cells appears to be an inactive form (Roberts and Sporn, 1990; Shirai et al, 1992, 1994; Bedossa et al, 1995). TGF-β1 may be activated with acidification, proteolysis or chaotropic agents (Roberts and Sporn, 1990; Bedossa et al, 1995). In this study, we have detected an active form of TGF-β1 by acidification in the urine of patients with HCC.

HCC appears to be associated with hepatitis B and C virus infection and is common in patients with cirrhosis caused by chronic viral hepatitis (Jeng and Tsai, 1991; Sherlock and Dooley, 1993; Tsai et al, 1993, 1994*a–f*, 1995, 1996*a,b*, 1997*a*). Thus, early detection of HCC in cirrhotic patients is important. During recent years, various serological markers have been developed in the diagnosis of HCC (Maussier et al, 1990; Tsai et al, 1990, 1991, 1995; Fujiyama et al, 1992; Sherlock and Dooley, 1993; Chuang et al, 1994; Colombo, 1995). Serum AFP is one of the most intensively studied tumour markers. By ROC curve analysis, the normal AFP is 5.2 ng $ml^{-1}$ (Massier et al, 1990). In cirrhotic patients with AFP values higher than 18.5 ng $ml^{-1}$, the likelihood of HCC being present is 95% (Massier et al, 1990). Previously, we suggested a cut-off value of 120 ng $ml^{-1}$, determined by ROC curve analysis, for diagnosis of HCC in cirrhotic liver (Tsai et al, 1995). The diagnostic cut-off value of AFP for HCC is 400 ng $ml^{-1}$ (Colombo, 1995). As shown in this study, AFP levels less than 400 ng $ml^{-1}$ were noted in 52.1.% (49/94) of HCC patients at the time of tumour detection. Furthermore, at least one-third of small HCCs and up to 30% of advanced HCC will be missed unless other diagnostic tools are used (Sherlock and Dooley, 1993; Colombo, 1995; Tsai et al, 1995, 1997*c,d*). In addition, AFP may be elevated in non-malignant liver disease (Sherlock and Dooley, 1993; Colombo, 1995; Tsai et al, 1995). It is obvious that AFP alone is not a reliable indicator for the detection of HCC in patients with a low AFP value. Therefore, additional and more sensitive diagnostic tools must be sought.

Based on the significant difference in TGF-β1 level between patients with cirrhotic HCC and patients with cirrhosis alone, and the close association between TGF-β1 level and development of HCC, an attempt was made to differentiate HCC from cirrhosis by TGF-β1. For clinical decision-making, the selected cut-off value of a laboratory test should provide the best diagnostic performance for either ruling out or ruling in the particular disease. The ROC curve analysis is a graphic method that can be used to determine this optimal cut-off level. In addition, it is a precise and valid measure of diagnostic accuracy (Swets, 1988). The calculated area under ROC curve of AFP (0.801) and TGF-β1 (0.730) in this study are between 0.7–0.9, which indicates that both accuracies are useful for diagnostic purposes (Swets, 1988). Based on the selected optimal cut-off value by ROC curve analysis, both TGF-β1 and AFP showed a good specificity, moderate sensitivity and high positive likelihood ratio (Table 4). There was no significant difference between their diagnostic accuracies. However, determination of AFP and TGF-β1 in parallel significantly improved the diagnostic accuracy and sensitivity without essentially decreasing the specificity (Table 4). Although each test may not have sufficient sensitivity, the simultaneous use of both tests may be highly discriminatory in the detection of HCC. However, parallel

© Cancer Research Campaign 1997

detection of both markers increases the number of tests performed, which must have cost implications. So, we suggest that assay for urinary TGF-β1 should be performed to improve the detection of HCC with low AFP production.

AFP is an oncofetal protein produced by HCC. Although the AFP gene was re-expressed in hepatoma cells, TGF-β1 may repress the AFP gene expression in hepatoma cells (Nakao et al, 1991). Our results also show a reverse relationship between levels of serum AFP and urinary TGF-β1. The urinary TGF-β1 level in HCC patients with normal AFP level was statistically higher than that in patients with raised AFP. This significantly inverse trend still existed even when a higher cut-off value of AFP (100 or 400 ng $ml^{-1}$) was used (data not shown). This observation also favours the use of urinary TGF-β1 as a complementary tumour marker for the detection of HCC in AFP-non-producing tumours.

As TGF-β1 is a major fibrogenic factor, an increase of urinary TGF-β1 might be expected in patients with active cirrhosis (with necroinflammatory histological features) by comparison with non-active cirrhosis in the absence of HCC (Castilla et al, 1991; Shrai et al, 1994; Bissell and Maher, 1996). In our patients with cirrhosis alone, there is an association between raised TGF-β1 level and worsening Child–Pugh grades. Our result supports the previous observation that TGF-β1 correlates with disease activity in cirrhosis (Castilla et al, 1991; Nagy et al, 1991). However, such elevations may have an important effect upon the specificity of the tests. In this study, urinary TGF-β1 levels greater than the selected cut-off points were found in 53.1% (50/94) of patients with HCC, 1.1% (1/94) of patients with cirrhosis alone (Tables 3 and 4) and none of the healthy controls (data not shown). It is of note that the only patient with cirrhosis alone and TGF-β1 level above the cut-off value is a patient with Child–Pugh C. In addition, elevation of AFP may be seen in patients with 'active' liver disease (Sherlock and Dooley, 1993; Tsai et al, 1994*b*, 1995; Colombo, 1995). On the other hand, the major aim of ideal tumour marker estimation in HCC is as a means of early detection (surveillance), particularly in the higher risk group. The present analysis has looked at a population of patients with a histologically proven diagnosis of HCC. It may be assumed that many of these had advanced disease and thus a high proportion would have significantly elevated tumour marker levels. The high specificity and sensitivity attained might therefore be overestimating the value of these tests as a surveillance tool. For example, although the median level of TGF-β1 in small HCCs (< 3 cm) was lower than the selected cut-off value (Table 2), 42.1% (8/19) of patients with small HCCs had TGF-β1 levels greater than the selected cut-off value. This observation suggests the potential of TGF-β1 in the early diagnosis of small HCCs. However, as the number of patients with small HCCs is low, whether TGF-β1 is actually useful for early detection of HCC requires further evaluation.

In conclusion, this study shows that urinary TGF-β1 level increases in patients with cirrhotic HCC. Raised urinary TGF-β1 level is closely associated with development of HCC. The addition of an assay for TGF-β1 to that for AFP gives a significant improvement in detection of HCC with low AFP production.

### ACKNOWLEDGEMENT

This study was supported by a grant from the National Science Council of the Republic of China (NSC 82-0412-B-037-003).

### REFERENCES

Baldwin GS and Zhang QX (1992) Measurement of gastrin and transforming growth factor alpha messenger RNA levels in colonic carcinoma cell lines by quantitative polymerase chain reaction. *Cancer Res* **52**: 2261–2267

Bedossa P, Peltier E, Terris B, Franco D and Poynard T (1995) Transforming growth factor-beta 1 (TGF-β1) and TGF-β1 receptors in normal, cirrhotic, and neoplastic human livers. *Hepatology* **21**: 760–766

Bissell DM and Maher JJ (1996) Hepatic fibrosis and cirrhosis. In *Hepatology: A Textbook of Liver Disease* 3rd edn, Zakim D and Boyer TD (eds), pp. 506–525. WB Saunders: Philadelphia

Castilla A, Prieto J and Fausto N (1991) Transforming growth factor β 1 and α in chronic liver disease: effects of interferon alpha therapy. *N Engl J Med* **324**: 933–940

Chuang LY, Tsai JH, Yeh YH, Chang CC, Yeh HW, Guh JY and Tsai JF (1991) Epidermal growth factor-related transforming growth factors in the urine of patients with hepatocellular carcinoma. *Hepatology* **13**: 1112–1116

Chuang LY, Hon WC, Yang ML, Chang CC and Tsai JF (1994) Urinary epidermal growth factor receptor-binding growth factors in the tumors of the digestive tract. *Clin Biochem* **27**: 485–489

Colombo M (1995) Should patients with chronic viral hepatitis be screened for hepatocellular carcinoma? *Viral Hepatitis Rev* **1**: 67–75

Coupes BM, Newstead CG, Short CD and Brenchley PEC (1994) Transforming growth factor β 1 in renal allograft recipients. *Transplantation* **57**: 1727–1731

Fujiyama S, Isuno K, Yamasaki K, Sato T and Taketa K (1992) Determination of optimum cutoff levels of plasma des-gamma-carboxy prothrombin and serum alpha-fetoprotein for the diagnosis of hepatocellular carcinoma using receiver operating characteristic curves. *Tumor Biol* **13**: 316–323

Ito N, Kawata S, Tamura S, Takaishi K, Yabuuchi I, Matsuda Y, Nishioka M and Tarui S (1990) Expression of transforming growth factor-beta 1 mRNA in human hepatocellular carcinoma. *Jpn J Cancer Res* **81**: 1202–1205

Ito N, Kawata S, Tamura S, Takaishi K, Shirai Y, Kiso S, Yabuuchi I, Matsuda Y, Nishioka M and Tarui S (1991) Elevated levels of transforming growth factor-beta and its polypeptide in human hepatocellular carcinoma. *Cancer Res* **15**: 4080–4083

Jeng JE and Tsai JF (1991) Hepatitis C virus antibody in hepatocellular carcinoma in Taiwan. *J Med Virol* **34**: 74–77

Kew MC (1996) Tumors of the liver. In *Hepatology: A Textbook of Liver Disease*, 3rd edn, Zakin D and Boyer TD (eds), pp. 1513–1548. WB Saunders: Philadelphia

Lee GH, Merlino G and Fausto N (1992) Development of liver tumors in transforming growth factor α transgenic mice. *Cancer Res* **52**: 5162–5170

Maussier ML, Valenza V, Schinco G and Galli G (1990) AFP, CEA, CA19-9 and TPA in hepatocellular carcinoma. *Int J Biol Markers* **5**: 121–126

Nakao K, Nakata K, Mitsuoka S, Ohtsuru A, Ido A, Hatano M, Sato Y, Nakayama T, Shima M, Kusumoto Y, Koji T, Tamaoki T and Nagataki S (1991) Transforming growth factor β 1 differentially regulates α-fetoprotein and albumin in HuH-7 human hepatoma cells. *Biochem Biophys Res Commun* **174**: 1294–1299

Nagy P, Schaff Z and Lapis K (1991) Immunohistochemical detection of transforming growth factor-β 1 in fibrotic liver disease. *Hepatology* **14**: 269–273

Nishimura R, Okumura H, Noda K, Yasumitsu H and Umeda M (1986) High level of β type transforming growth factor activity in human urine obtained from cancer patients. *Jpn J Cancer Res* **77**: 560–567

Parkin DM, Stjernsward T and Muir CS (1984) Estimates of the worldwide frequency of twelve major cancers. *Bull World Health Organ* **62**: 163–182

Pugh RN, Murray-Lyon IM, Dawson JL, Peitroni MC and Williams R (1973) Transection of the esophagus for bleeding esophageal varices. *Br J Surg* **60**: 646–649

Ranganathan G, Lyons R, Jiang NS and Moses H (1987) Transforming growth factor β in normal human urine. *Biochem Biophys Res Comm* **148**: 1503–1512

Roberts AB and Sporn MB (1990) The transforming growth factor betas. In *Handbook of Experimental Pharmacology*, Vol. 95, Sporn MB and Roberts AB (eds), pp. 419–472 Springer: Heidelberg, Germany

Roberts AB, Thompson NL, Heine U, Flanders C and Sporn MB (1988) Transforming growth factor-beta: possible roles in carcinogenesis. *Br J Cancer* **57**: 594–600

Sherlock S (1994) Viruses and hepatocellular carcinoma. *Gut* **35**: 828–832

Sherlock S and Dooley J (1993) *Disease of the Liver and Biliary System* pp. 503–531. Blackwell Scientific: Oxford

Sherwin SA, Twardzik DR, Bohn WH, Cockley KD and Todaro GJ (1983) High-molecular-weight transforming growth factor activity in the urine of patients with disseminated cancer. *Cancer Res* **43**: 403–407

© Cancer Research Campaign 1997

Shirai Y, Kawata S, Ito N, Tamura S, Takaishi K, Kiso S, Tsushima H and Matsuzawa Y (1992) Elevated levels of plasma transforming growth factor-β in patients with hepatocellular carcinoma. *Jpn J Cancer Res* **83**: 676–679

Shirai Y, Kawata S, Tamura S, Ito N, Tsushima H, Takaishi K, Kiso S and Matsuzawa Y (1994) Plasma transforming growth factor-β1 in patients with hepatocellular carcinoma. *Cancer* **73**: 2275–2279

Simonetti RG, Camma C, Fiorello F, Politi F, D'Amico G and Pagliaro L (1991) Hepatocellular carcinoma: a worldwide problem and the major risk factors. *Dig Dis Sci* **36**: 962–972

Sox HC, Blatt MA, Higgins MC and Marton K (1989) *Medical Decision Making*, pp. 67–146. Butterworth: London

Swets JA (1988) Measuring the accuracy of diagnostic systems. *Science* **240**: 1285–1293

Tsai JF, Tsai JH and Chang WY (1990) Relationship of serum α-feto protein to circulating immune complexes and complements in patients with hepatitis B surface antigen-positive hepatocellular carcinoma. *Gastroenterol Jpn* **25**: 388-393

Tsai JF, Tsai JH, Chang WY and Ton TC (1991) Elevation of circulating immune complexes and its relationship to α-fetoprotein levels in patients with hepatitis B surface antigen-positive hepatocellular carcinoma. *Cancer Invest* **9**: 137–143

Tsai JF, Chang WY, Jeng JE, Wang LY, Hsieh MY, Chen SC, Chuang WL, Lin ZY and Tsai JH (1993) Hepatitis C virus infection as a risk factor for non-alcoholic liver cirrhosis in Taiwan. *J Med Virol* **41**: 296–300

Tsai JF, Chang WY, Jeng JE, Ho MS, Lin ZY and Tsai JH (1994*a*) Hepatitis B and C virus infection as risk factors for liver cirrhosis and cirrhotic hepatocellular carcinoma: a case–control study. *Liver* **14**: 98–102

Tsai JF, Chang WY, Jeng JE, Ho MS, Lin ZY and Tsai JH (1994*b*) Frequency of raised alpha-fetoprotein level among Chinese patients with hepatocellular carcinoma related to hepatitis B and C. *Br J Cancer* **69**: 1157–1159

Tsai JF, Chang WY, Jeng JE, Ho MS, Lin ZY and Tsai JH (1994*c*) Hepatitis B and C virus infection as risk factors for hepatocellular carcinoma in Chinese: a case–control study. *Int J Cancer* **56**: 619–621

Tsai JF, Jeng JE, Chang WY, Ho MS, Lin ZY and Tsai JH (1994*d*) Hepatitis C virus infection among patients with chronic liver disease in an area hyperendemic for hepatitis B. *Scand J Gastroenterol* **29**: 550–552

Tsai JF, Chang WY, Jeng JE, Ho MS, Lin ZY and Tsai JH (1994*e*) Effects of hepatitis C and B viruses infection on the development of hepatocellular carcinoma. *J Med Virol* **44**: 92–95

Tsai JF, Margolis HS, Jeng JE, Ho MS, Ko YC, Chang WY, Lin ZY and Tsai JH (1994*f*) Association between hepatitis B and C virus infection and Chinese hepatocellular carcinoma: a case–control study. In *Viral Hepatitis and Liver Disease*, Nishioka K, Suzuki H, Mishiro S and Oda . (eds), pp. 697–700. Springer: Tokyo

Tsai JF, Jeng JE, Chang WY, Ho MS, Lin ZY and Tsai JH (1995) Clinical evaluation of serum α-feto-protein and circulating immune complexes as tumor markers of hepatocellular carcinoma. *Br J Cancer* **72**: 442–446

Tsai JF, Jeng JE, Ho MS, Chang WY, Hsieh MY, Lin ZY and Tsai JH (1996*a*) Independent and additive effect modification of hepatitis C and B viruses infection on the development of chronic hepatitis. *J Hepatol* **24**: 271–276

Tsai JF, Jeng JE, Ho MS, Chang WY, Hsieh MY, Lin ZY and Tsai JH (1996*b*) Additive effect modification of hepatitis B surface antigen and e antigen on the development of hepatocellular carcinoma. *Br J Cancer* **73**: 1498–1502

Tsai JF, Jeng JE, Ho MS, Wang CS, Chang WY, Hsieh MY, Lin ZY and Tsai JH (1997*a*) Serum alanine aminotransferase level in relation to hepatitis B and C virus infection among blood donors. *Liver* (in press)

Tsai JF, Jeng JE, Chaung LY, Chang WY and Tsai JH (1997*b*) Urinary transforming growth factor-β1 as a predictor of hepatitis C virus-related chronic liver disease: correlation between high levels and severity of disease. *Hepatology* (in press)

Tsai JF, Chaung LY, Ho MS, Chang WY, Hsieh MY, Lin ZY and Tsai JH (1997*c*) Urinary transforming growth factor-β1 in relation to serum α-fetoprotein in hepatocellular carcinoma. *Scand J Gastrenterol* (in press)

Tsai JF, Chaung LY, Jeng JE, Yang ML, Ho MS, Chang WY, Hsieh MY, Lin ZY and Tsai JH (1997*d*) Clinical relevance of transforming growth factor-β1 in the urine of patients with hepatocellular carcinoma. *Medicine* (in press)

Yeh YC, Tsai JF, Chuang LY, Yeh HW, Tsai JH, Florine DL and Tam JP (1987) Elevation of transforming growth factor α and its relationship to the epidermal growth factor and α-fetoprotein levels in patients with hepatocellular carcinoma. *Cancer Res* **47**: 896–901

© Cancer Research Campaign 1997